# Nurse teachers’ knowledge about epilepsy and communication issues between schools and medical institutions: A nationwide questionnaire survey in Japan

**DOI:** 10.1002/epi4.12390

**Published:** 2020-04-12

**Authors:** Kiyohito Terada, Yushi Inoue, Takuji Nishida, Daisuke Mishiro, Mitsuhiko Yamano, Tomoo Aoyagi, Yuji Tadokoro

**Affiliations:** ^1^ Department of Epileptology NHO Shizuoka Institute of Epilepsy and Neurological Disorders Shizuoka Japan; ^2^ Nonprofit Organization Chichinpuipuiakebono Oita Japan; ^3^ Department of Neurology Tokai University Isehara Japan; ^4^ Japan Epilepsy Association Tokyo Japan

**Keywords:** children with epilepsy, education, modified grounded theory approach, network, student

## Abstract

**Objective:**

The importance of school teachers’ knowledge of and attitudes toward epilepsy and the communication between educational and medical systems is widely appreciated, but exploration of these factors in Japan has been extremely limited. In order to identify issues in support systems for students with epilepsy and bridge the gaps in communication between schools and medical institutions in Japan, we performed a nationwide questionnaire survey of nurse teachers (nurses in charge of health education/care at schools).

**Methods:**

We mailed a questionnaire to 900 nurse teachers all over Japan. It included six items on general epilepsy knowledge and 15 items on information about each student with epilepsy in their schools. We used a modified grounded theory approach (M‐GTA) to analyze open‐ended questions.

**Results:**

We received responses from 640 (71.1%) nurse teachers. In their schools, there were 237 253 students, of whom 1565 had epilepsy. Most nurse teachers (84.7%) understood that epilepsy is a neurological disease. When performing first aid for a seizure, they would observe the seizure calmly (85.9%) and/or secure the airway (75.3%). There were 1398 responses about individual students with epilepsy (89.3%). Nurse teachers knew the seizure type in 70.0% of these students, seizure frequency in 76.8%, triggers in 38.9%, and appropriate first aid for 79.0%. Some nurse teachers (30.2%) obtained information on students with epilepsy from medical institutions. They knew more about their students’ seizures than those without medical information. Existing forms for communicating information on students with epilepsy between schools and physicians were not actively utilized. Responses to open questions converged on safety at school.

**Significance:**

Japanese nurse teachers understand epilepsy relatively well, but do not fully grasp the condition of each student with epilepsy. Better information flow from medical institutions is needed. Active communication is necessary to support the safety of students with epilepsy at school.

**Key point**

- We conducted Japan's first nationwide questionnaire survey of nurse teachers.
- Many nurse teachers had good general knowledge of epilepsy and understood the clinical information about their students with epilepsy.
- However, information from medical institutions would improve their knowledge about students with epilepsy.
- Better communication is needed between medical institutions and schools.
- Nurse teachers’ responses to open questions converge on safety issues and not to future careers of students with epilepsy.

## 1 INTRODUCTION

The knowledge of and attitudes toward epilepsy among school teachers have been intensively investigated in many countries and in systematic reviews.^1^, ^2^ These studies demonstrated that teachers have poor knowledge about epilepsy, and when the teachers’ knowledge is poor, attitudes toward students with epilepsy are negative. It was also reported that poor knowledge and attitudes are associated with students with epilepsy having accidents^3^ and that education on epilepsy improved the teachers’ knowledge and their ability to manage their students’ seizures.^4^ A large‐scale questionnaire survey of school teachers examining their knowledge of and attitudes toward epilepsy was recently conducted in Japan.^5^ Teachers scored poorly in general questions about epilepsy and showed a negative attitude in some responses.^5^ However, any further exploration of the knowledge and attitudes of teachers in Japan has been limited.^6^, ^7^ Furthermore, although communication between educational and medical systems is understood to be important, it was found to be inadequate.^8^ Japanese teachers expressed an interest in communicating with medical professionals about students with epilepsy,^7^ but the network between educational and medical professionals remains insufficient.

In Japan, education is mandatory until the end of junior high school. There are several systems in place to support children with disabilities. For those who have difficulty in learning in a general education school (GES), special education schools (SES) are available. Available guidelines on the medical needs of children in school with disabilities include the *School life guidance and management form* from the Japan Society of School Health (http://www.hokenkai.or.jp/en/) and, specifically for students with epilepsy, the *List for guidance of daily life in children with epilepsy*.^9^ Furthermore, Japanese law requires that all primary and junior high schools should have at least one nurse teacher who is a specially qualified nurse in charge of health care, health consultation, health education, and first aid in school. Nurse teachers do not usually have classes, although they provide health education to students in cooperation with other teachers. The nurse teacher is a key person who plays an important role in the school support system for students with epilepsy.

In this study, we conducted a nationwide questionnaire survey of nurse teachers, aiming to clarify issues of support systems for students with epilepsy and bridge the gaps in communication between schools and medical institutions.

## 2 METHODS

This cross‐sectional study was conducted among nurse teachers in elementary and junior high schools in Japan, in collaboration with the National Liaison Committee of Yogo Teacher (NLCYT). We mailed 900 questionnaires to nurse teachers, to be completed anonymously. As NLCYT has 45 branches nationwide, 20 nurse teachers were selected from 10 elementary and 10 junior high schools in each branch, with one SES expected to be involved in each branch.

The questionnaire included six items on general knowledge about epilepsy and 15 items on information about each student with epilepsy in their school. The content of the questionnaire was developed by epileptologists, a clinical psychologist, an employment counselor, and members of a patients’ self‐help group (together, the authors) with reference to previous studies in Japan^6^, ^7^ and with advice from nurse teachers (members of NLCYT) and the investigative agency. The authors judged the appropriateness of the answers if there were any doubts.

Non‐parametric statistical analysis was performed using the chi‐square test and Mann‐Whitney *U* test. In the chi‐square test, when observed values were zero in both compared cells, these cells were excluded from the analysis. When the chi‐square comparison demonstrated a statistically significant difference between groups, a residual analysis was applied to identify the specific cells making the greatest contribution to the difference. Excel TOUKEI 7.0 (Tokyo, ESUMI Co., Ltd.) was used for these analyses.

The experimental protocol was approved by the Ethical Committee of the NHO Shizuoka Institute of Epilepsy and Neurological Disorders (2017‐2018).

The questionnaire contained four semi‐structured open questions. We expected each nurse teacher to answer these questions according to their knowledge and experience. Therefore, a social constructivist modified grounded theory approach (M‐GTA) was applied to analyze the answers^10^ as follows:

1. KH coder^11^, ^12^ was used to pick up words and classify them preliminarily.
2. Analysis worksheets with four columns (examples, theoretical notes, concept definition, and concept name) were used to generate concepts. The preliminarily classified words were recorded in the “examples” column; then, the interpretations of the words were recorded in the “theoretical notes” column.
3. Comparative analysis was performed to examine other similar and contrasting examples.
4. For coding, after finding a common meaning among examples, the concept definition and the concept name were described in a short statement.
5. After generating multiple concepts, the inter‐concept relationships were examined to clarify their directionality and generate a category under which these concepts fall.
6. A schematic diagram of the relationships between concepts and categories was developed, and then, a core category was formulated.
7. When no additional concepts could be generated and when available concepts and categories were deemed sufficient to explain the phenomena, this indicated that theoretical saturation had been reached.

## 3 RESULTS

### 3.1 Nurse teachers’ general knowledge about epilepsy

Table 1 summarizes the answers to the general knowledge questions about epilepsy. Of 900 nurse teachers who were sent the questionnaire, 640 (602 from GESs, 34 from SESs, and four from other schools) returned the questionnaires (response rate: 71.1%). In their schools, there were 237 253 students (1689 in SESs and 3293 in other schools), of whom 1565 had epilepsy (332 in SESs and 89 in other schools).

**Table 1:**
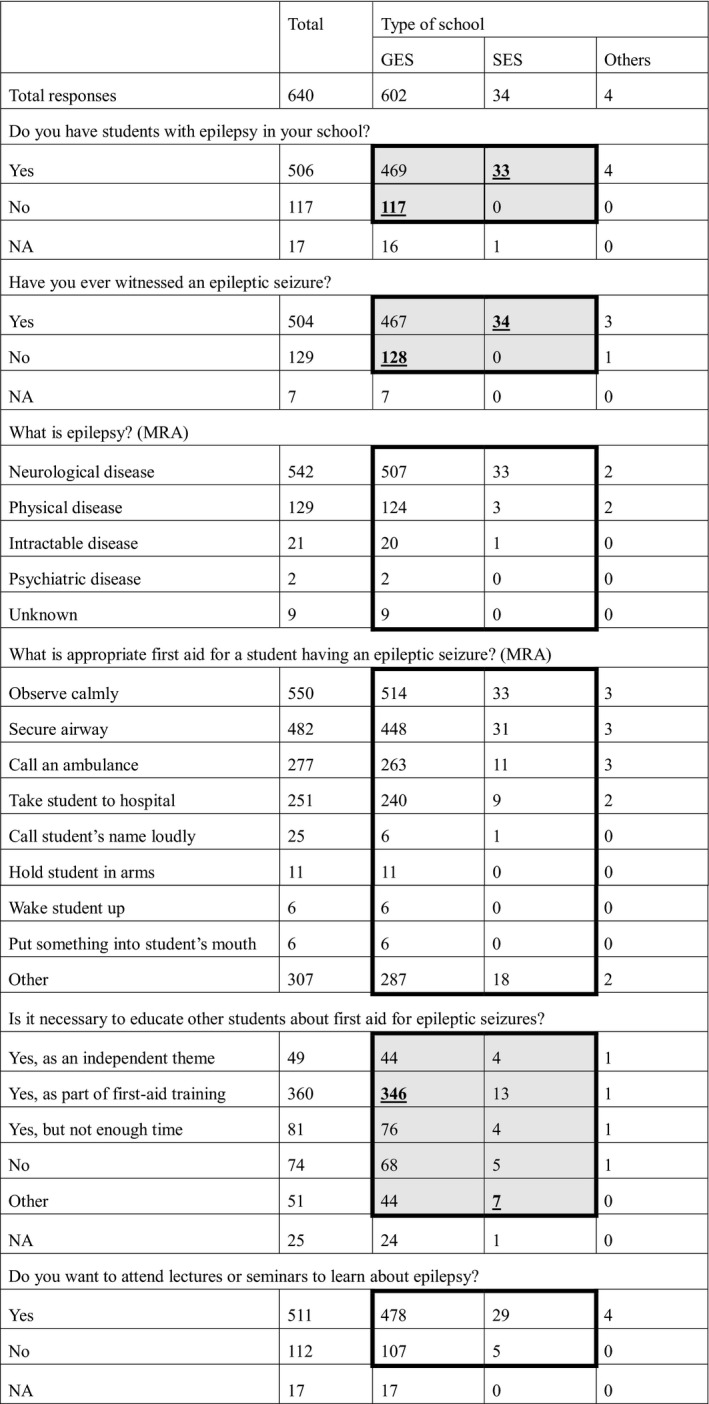
Nurse teachers’ general knowledge about epilepsy

Note

Thick outline indicates that statistical analysis was performed in the box. Shaded cells indicate a statistically significant difference within the box. Bold, underlined numbers indicate significantly higher results in the residual analysis after the chi‐square test.

Abbreviations: GES, general educational school; MRA, multiple responses allowed; NA, no answer; SES, special educational school.

Of 602 nurse teachers in GESs, 469 had students with epilepsy and 117 did not; of 34 nurse teachers in SESs, 33 had students with epilepsy. There were significantly more students with epilepsy in SESs than in GESs (*P* = .004). Most (467) nurse teachers in GESs and all 34 in SESs had witnessed an epileptic seizure, although 128 nurse teachers in GESs had not, meaning that significantly more nurse teachers in SESs than in GESs had witnessed a seizure (*P* = .002).

Epilepsy was recognized as a neurological disease by 542 nurse teachers, a physical disease by 129, an intractable disease by 21, and a psychiatric disease by 2. For these understandings of epilepsy, there were no differences between GESs and SESs (*P* = .47).

For questions about first aid, 550 nurse teachers responded that they would calmly observe the seizure, 482 would secure the airway, 277 would call an ambulance, 251 would take the student to hospital, 25 would call the student's name loudly, 11 would hold them in their arms, six would attempt to wake them up, six would put something into the student's mouth, and 307 responded “other,” which included calling the student's parents, keeping the student safe, observing the seizure carefully, controlling the other students, and doing as indicated by a physician. There were no differences between responses from nurse teachers at GESs and those at SESs (*P* = .69).

Regarding the need for education about first aid for students having a seizure, 81 nurse teachers answered “necessary but there is not enough time,” 49 answered “necessary as an independent theme,” 360 answered “necessary as part of first aid training for various emergency cases,” and 74 answered “unnecessary.” More nurse teachers in GESs than in SESs responded that it is necessary as part of general first aid training (*P* = .036).

To the question whether they want to attend lectures or seminars to learn about epilepsy, 511 nurse teachers answered yes, with no difference between those at GESs and those at SESs (*P* = .60). The content of such lectures requested by nurse teachers was rather simple, such as first aid for epileptic seizures, fundamental knowledge of epilepsy, limitations that students with epilepsy face in daily living, and treatment of epilepsy.

To clarify the relationship between knowledge and experience of epilepsy, we compared data between nurse teachers who had students with epilepsy in their school and those who did not, as well as those who had witnessed epileptic seizures and those who had not, but there were no significant differences (Table S1).

### 3.2 Nurse teachers’ recognition of seizures in individual students

Our respondents reported individual information on 1398 out of 1565 students with epilepsy (response rate: 89.3%) (1149 in GESs, 207 in SESs, and 42 in other schools). From these data, the prevalence of epilepsy was calculated as 0.49% in GESs and 19.66% in SESs.

Information was obtained from medical institutions by 422 nurse teachers (Inf+), whereas 964 did not seek such information (Inf−). Significantly more nurse teachers in SESs obtained information than those in GESs (*P* < .001; Table 2). Comorbidity data for students with epilepsy are shown in Table S2.

**Table 2:**
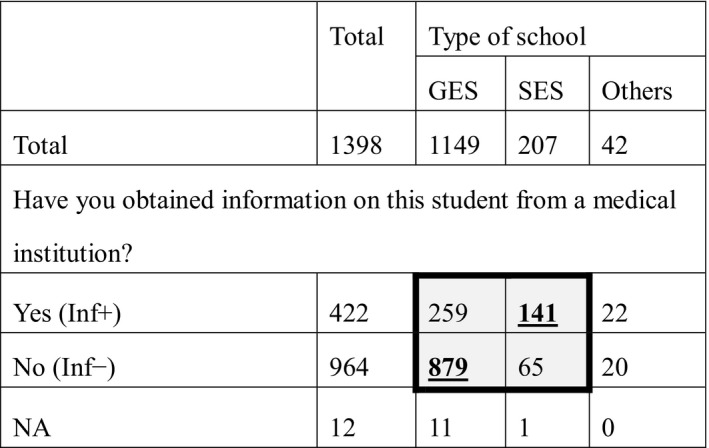
Information on individual students with epilepsy

Note

Thick outline indicates that statistical analysis was performed in the box. Shaded cells indicate a statistically significant difference within the box. Bold, underlined numbers indicate significantly higher results in the residual analysis after the chi‐square test.

Abbreviations: GES, general educational school; Inf−, information from medical institution not obtained; Inf+, information from medical institution obtained; NA, no answer; SES, special educational school.

**FIGURE 1:**
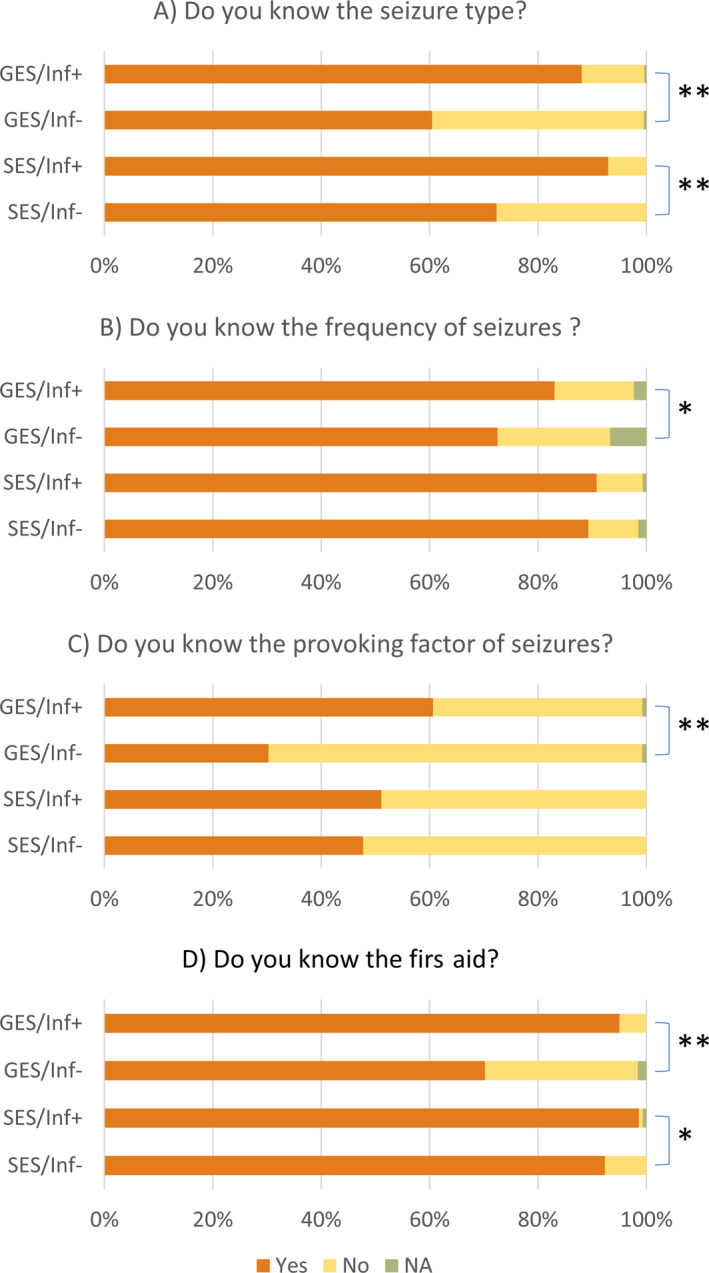
Relationship between information from medical institutions and responses to each question. GES, general education school; SES, special education school; Inf+, information from medical institution obtained; Inf−, information from medical institution not obtained; NA, no answer. *P < .05, **P < .005

Nurse teachers knew the seizure types of 978 students, but not of 415 students. Those in SESs knew students’ seizure types more frequently than those in GESs (*P* < .001; Table 3). Inf+ nurse teachers knew the seizure types more frequently than Inf− nurse teachers in both GESs and SESs (*P* < .001 and *P* < .001, respectively; Table 3, Figure 1A).

**Table 3:**
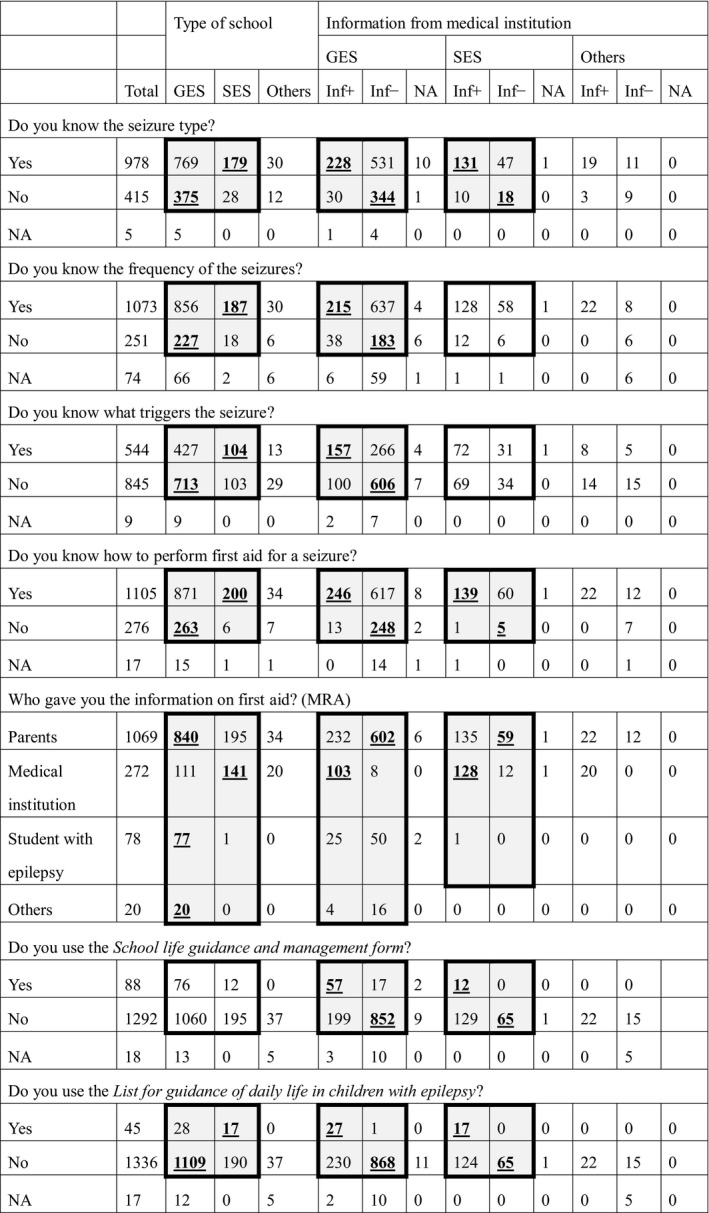
Nurse teachers’ knowledge of individual students’ seizures

Note

Thick outline indicates that statistical analysis was performed in the box. Shaded cells indicate a statistically significant difference within the box. Bold, underlined numbers indicate significantly higher results in the residual analysis after the chi‐square test.

Abbreviations: GES, general educational school; Inf−, information from medical institution not obtained; Inf+, information from medical institution obtained; MRA, multiple responses allowed; NA, no answer; SES, special educational school.

Nurse teachers knew the seizure frequency of 1073 students with epilepsy. More knew this information in SESs than in GESs (*P* < .001), and more Inf+ nurse teachers knew this than Inf− nurse teachers in GESs (*P* < .01) but not in SESs (*P* = .85; Table 3, Figure 1B). Seizure type and frequency data are shown in Table S3.

Nurse teachers knew seizure triggers for 544 students, but not for 845. More were aware of triggers in SESs than in GESs (*P* < .001), and more Inf+ than Inf− in GESs (*P* < .001) but not in SESs (*P* = .65; Table 3, Figure 1C).

For 1105 students with epilepsy, nurse teachers had already checked how to administer first aid when a seizure occurred, more in SESs than in GESs (*P* < .001) and more frequently when Inf+ than when Inf− in both GESs and SESs (*P* < .001 and .006, respectively; Table 3, Figure 1D).

**FIGURE 2:**
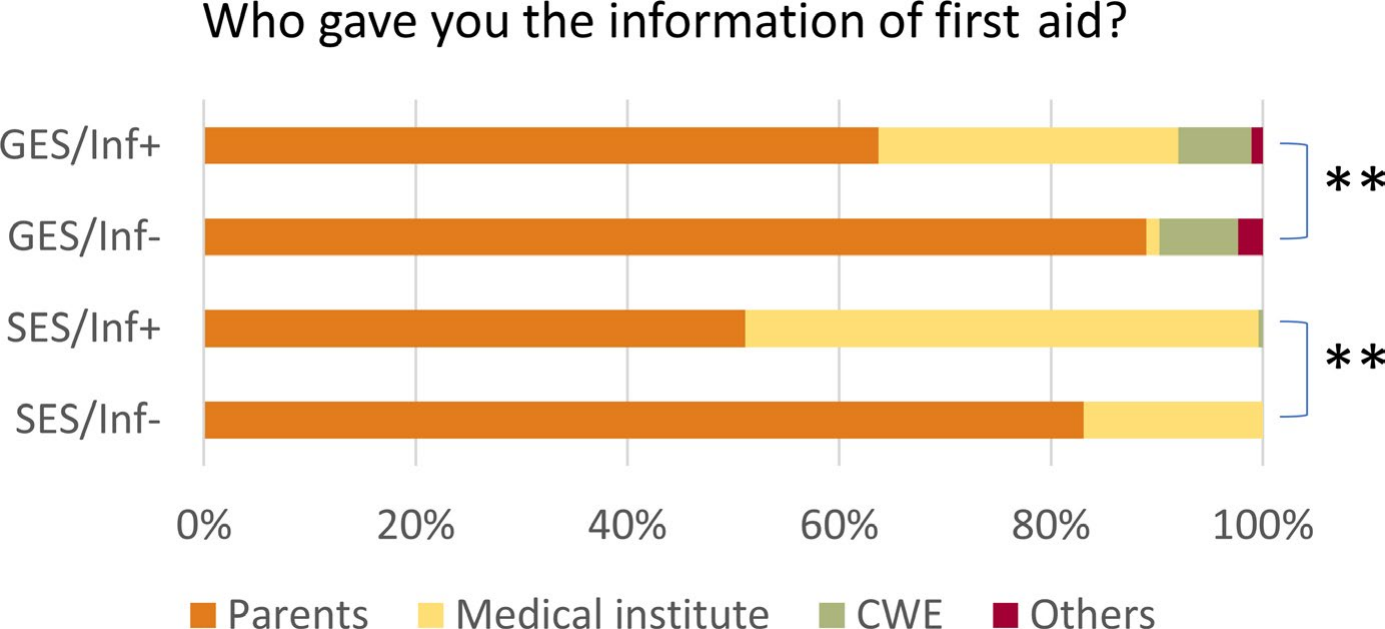
Relationship between information from medical institutions and source of information about first aid. GES, general education school; SES, special education school; Inf+, information from medical institution obtained; Inf−, information from medical institution not obtained; NA, no answer. *P < .05, **P < .005

The source of medical information was parents for 1069 students with epilepsy, medical institutions for 272, the student themselves in 78 cases, and other sources in 20 cases. More nurse teachers in SESs than in GESs obtained information from medical institutions (*P* < .001). Information from medical institutions was used reliably in both GESs and SESs (*P* < .001 and <.001, respectively; Table 3, Figure 2).

### 3.3 Communication between teachers and physicians

Of the existing forms by which teachers and physicians could communicate about students with epilepsy, the *School life guidance and management form* was used for only 6.2% of these students and the *List for guidance of daily life in children with epilepsy* for 3.2% (Table 3). The *List for guidance* form was used more in SESs than in GESs (*P* < .001), but there was no difference in the use of the *School life guidance* form between the two types of schools (*P* = .63). Both forms were used more frequently by Inf+ nurse teachers than Inf− nurse teachers in GESs (*List for guidance* form, *P* < .001; *School life guidance* form, *P* = .015) and SESs (*P* < .001 and .004, respectively). Nurse teachers indicated a lack of items on these forms, such as descriptions of seizures, frequency of seizures, first aid, monitoring systems, drug information, and restriction in school life, and proposed that they should be added.

### 3.4 Responses to open questions

**FIGURE 3:**
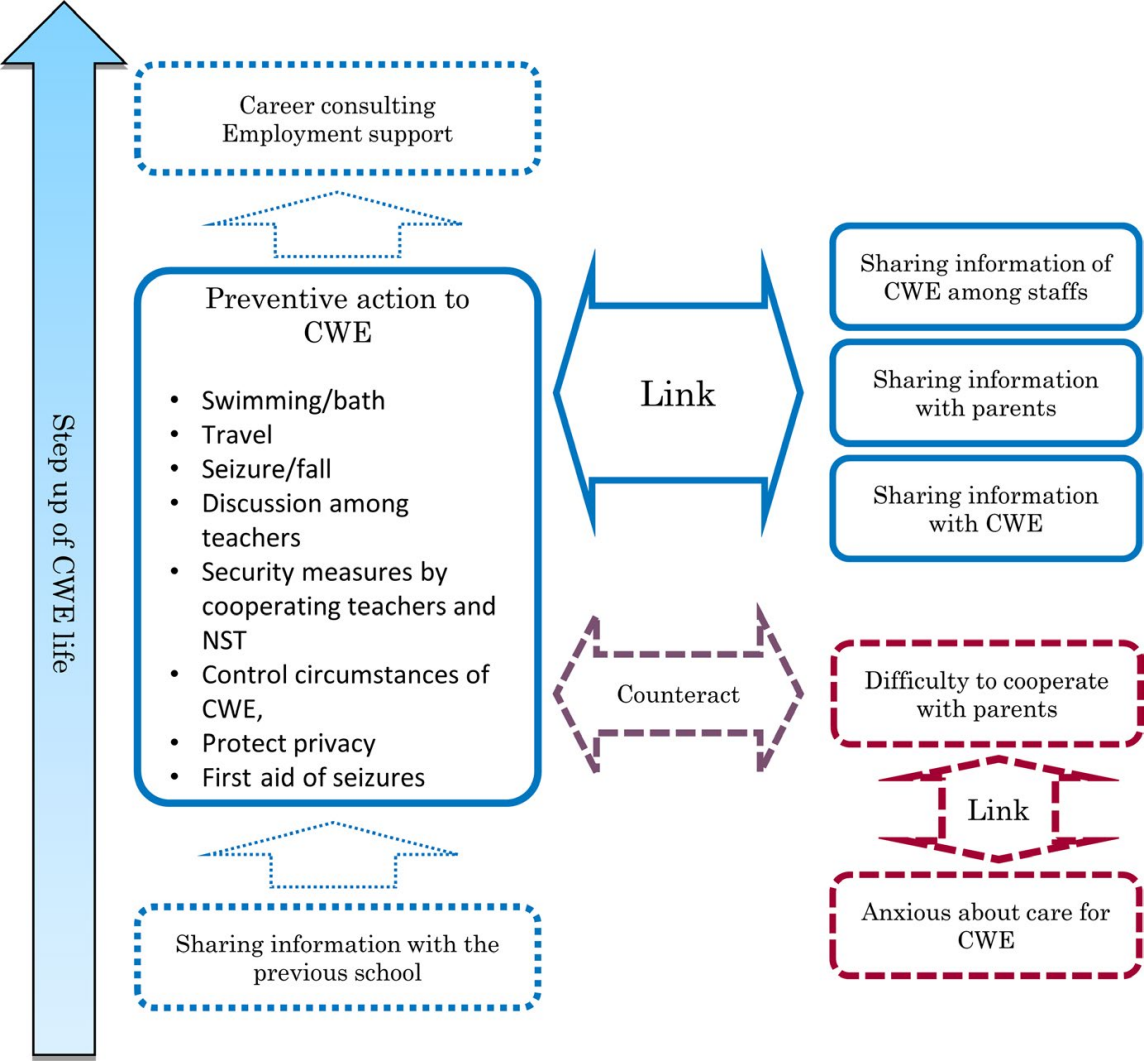
Schematic of relationship between concepts generated in M‐GTA. The main concept, “preventive actions for students with epilepsy,” is closely linked with other concepts such as “sharing of information on students with epilepsy among staff,” “sharing information with parents,” and “sharing information with students with epilepsy.” The main concept counteracts the concept “difficulty cooperating with parents” which links with “anxious about care for students with epilepsy.” There was no content in the nurse teachers’ responses about “career consulting” or “employment support,” despite this being the fundamental function of schools

A summary of the open questions is shown in Table S4. M‐GTA analysis (Figure 3) generated the main concept “preventive actions for students with epilepsy” and included the terms *swimming/bath*, *travel*, *seizure/fall*, *discussion among teachers*, *security measures by cooperating teachers and nurse teachers*, *control of circumstances of students with epilepsy*, *privacy protection*, and *seizure first aid*. The main concept linked with the concepts of “sharing of information on students with epilepsy among staff,” “sharing information with parents,” and “sharing information with students with epilepsy.” Another concept, “difficulty cooperating with parents,” linked with the concept “anxious about care for students with epilepsy” and counteracted the main concept. All these concepts converged to the safety of the students in school life; no responses or comments related to the next step after school, employment, and/or careers of the students.

## 4 DISCUSSION

Although the teachers’ knowledge of and attitudes toward epilepsy have been extensively examined worldwide, there have been few reports from Japan.^5^, ^6^, ^7^ Previous studies showed that education about epilepsy improved the knowledge of teachers and their management of students’ seizures,^4^ and that the network between educational and medical systems is important but currently inadequate.^8^

This is the largest survey of nurse teachers conducted in Japan. We investigated how knowledgeable they are about epilepsy and individual students with epilepsy, and examined the support network around these students, paying particular attention to the school type (GES/SES) in which the nurse teachers work and the information from medical institutions.

### 4.1 Prevalence of epilepsy

Our questionnaire revealed a prevalence of 0.49% in GESs and 19.66% in SESs. In 2017, there were 30 420 GESs (9 781 992 students) and 1135 SESs (71 802 students) in Japan. Therefore, the estimated prevalence in this age group is 0.63%. Because nurse teachers might not be familiar with each student with epilepsy, the real prevalence could be higher than this.

### 4.2 Differences between GES and SES

In Japan, children who have difficulty studying in GESs due to a disability can study in SESs. Because we suspected that nurse teachers’ knowledge of epilepsy, and the support network around students with epilepsy, would differ between GESs and SESs, we analyzed these two school types separately.

The basic knowledge about epilepsy was not different between nurse teachers in SESs and in GESs. However, there were significant differences between school types in the information nurse teachers understood on each student with epilepsy. Most nurse teachers, in both GESs and SESs, had learned about epilepsy during their career, but those in SESs may have had more practical experience and need to pay more attention to children's health conditions than those in GES.

### 4.3 Nurse teachers’ general knowledge about epilepsy

In the present study, 84.7% of nurse teachers thought epilepsy is a neurological disease and 0.3% that it is a psychiatric disease. In 1983, Ono and Makino surveyed 229 teachers in six GESs and one SES^6^ and demonstrated that 29.5% of teachers thought epilepsy is hereditary. Miyake surveyed 25 GESs and 11 SESs (1335 teachers) twice in 1978 and 1990,^7^ reporting that 22.9% of teachers in 1978 and 17.0% in 1990 thought epilepsy is hereditary, and 8.5% in 1978 and 4.1% in 1990 thought epilepsy is a psychiatric disease. These improvements in knowledge may be due not only to a change over time but also to differences between teachers and nurse teachers.

Teachers with personal experience of epilepsy were previously reported to have a greater knowledge of epilepsy^13^ than those who had not witnessed or experienced it first‐hand. However, we found no significant differences in knowledge of epilepsy between nurse teachers who had students with epilepsy in their school and those who had none, and between nurse teachers who had witnessed epileptic seizures and those who had not. We expect that this difference in results from those previously reported is because we surveyed nurse teachers who had already had a basic knowledge of epilepsy.

### 4.4 Communication between teachers and physicians

Nurse teachers obtained information from medical institutions for 30.2% of students with epilepsy. Those in SESs obtained information from medical institutions more frequently (68.1%) than those in GESs (22.5%). In the previous reports from Japan, Ono and Makino reported that 57.1% of teachers in SESs, but only 3%‐10% in GESs, discussed students with epilepsy with a doctor.^4^ Miyake reported that 8.5% of teachers obtained information from doctors in 1978, rising to 17.6% in 1990.^5^ This indicates that the communication between educational and medical institutions in Japan is improving.

### 4.5 Impact of information from medical institutions

Our survey revealed that obtaining information from medical institutions significantly improved nurse teachers’ knowledge on each student with epilepsy, including seizure type, frequency, triggers, and appropriate first aid. These improvements were observed in both GESs and SESs. Because such information is expected to help nurse teachers support students with epilepsy, communication between educational and medical systems should be a priority for improving the school life of students with epilepsy.

### 4.6 Communication between teachers and physicians

In Japan, it is difficult for nurse teachers and physicians to be in direct contact because of issues of personal information protection. Our survey revealed that, at least for first aid, information was provided mainly by parents (76.4%). Therefore, parents seem to be a good intermediary via which nurse teachers and physicians can communicate. Two forms already exist for this purpose: the *School life guidance and management form* from the Japan Society of School Health and the *List for guidance of daily life in children with epilepsy*.^9^ However, our survey showed that very few nurse teachers use these forms (6.2% and 3.2%, respectively); in addition, some nurse teachers (34.1% and 20.0%, respectively) considered these forms inadequate and requested the addition of several more items. We recommend that these forms be revised and redistributed.

### 4.7 Education about first aid for epileptic seizures

It was previously shown that epilepsy education could improve knowledge and attitudes toward epilepsy,^4^ reinforcing the importance of public education in reducing the stigma of epilepsy.^1^ Education about first aid for epileptic seizures is very useful, not only for practical purposes but also as way of raising awareness about the condition. In this survey, 75.5% of nurse teachers believed that their students should be taught to administer first aid for epileptic seizures. Furthermore, 79.8% expressed a desire to learn about epilepsy themselves. We recommend implementing various materials and/or methods for educating and raising awareness about epilepsy for everyone in the school, including children, teachers, and nurses.

### 4.8 Systematic analysis of open questions

Open questions are very important for clarifying the underlying thinking that multiple‐choice questions cannot identify. However, they are not easy to analyze systematically. We used M‐GTA, which revealed that the comments from nurse teachers on students with epilepsy mainly converged to issues of the students’ safety in school life, with none relating to the next stage after school, employment, and/or career of these students. This could be explained by the fundamental duty of nurse teachers being the students’ health care and first aid. However, because school is the place where children study for their future (independence, employment, and/or public participation), and because some people with epilepsy have difficulty getting jobs because of low self‐esteem, education of students with epilepsy should also account for their future and aim to improve their self‐esteem in school life.

### 4.9 Limitations

The response rate to our survey was relatively high (we received responses from 71.1% of nurse teachers on 89.3% of students with epilepsy). However, this study may still suffer from non‐response bias because nurse teachers who do not understand epilepsy may have refused to respond, resulting in an overestimation of nurse teachers’ knowledge about epilepsy. Furthermore, some students might not have informed their nurse teachers that they have epilepsy. Even if nurse teachers obtained information from medical institutions, the quality of information may vary between epilepsy specialists and non‐specialists. However, despite the potential bias and confounders inherent to such surveys, our large‐scale nationwide study is nevertheless valuable for revealing the issues in the support systems for students with epilepsy and highlights the importance of communication between educational and medical professionals.

## CONFLICTS OF INTEREST

None of the authors has any conflict of interest to disclose. We confirm that we have read the Journal's position on issues involved in ethical publication and affirm that this report is consistent with those guidelines.

## Supporting information

Table S1‐S4
