# Supplementary material for: Nurse teachers’ knowledge about epilepsy and communication issues between schools and medical institutions: A nationwide questionnaire survey in Japan
Source: Epilepsia Open. 2020 Apr 12;5(2):220–9. doi: 10.1002/epi4.12390 (PMC7278569; doi:10.1002/epi4.12390)
Supplement: Supplementary file 1 — Table S1‐S4 [file EPI4-5-220-s001.docx]

**Supplement**

Suppl Table 1: Nurse teachers’ general knowledge about epilepsy (detailed data)

|  |  | Type of school | | | Students with epilepsy in school | | | Witnessed seizures | | |
| --- | --- | --- | --- | --- | --- | --- | --- | --- | --- | --- |
|  | Total | GES | SES | Others | Yes | No | NA | Yes | No | NA |
| Total response | 640 | 602 | 34 | 4 |  |  |  |  |  |  |
| #1. Do you have students with epilepsy in your school? | | | | | | | | | | |
| Yes | 506 | 469 | **33** | 4 |  |  |  |  |  |  |
| No | 117 | **117** | 0 | 0 |  |  |  |  |  |  |
| NA | 17 | 16 | 1 | 0 |  |  |  |  |  |  |
| #2. Have you ever witnessed an epileptic seizure? | | | | | | | | | | |
| Yes | 504 | 467 | **34** | 3 | 402 | 89 | 13 |  |  |  |
| No | 129 | **128** | 0 | 1 | 97 | 28 | 4 |  |  |  |
| NA | 7 | 7 | 0 | 0 | 7 | 0 | 0 |  |  |  |
| #3. What is epilepsy? (MRA) | | | | | | | | | | |
| Neurological disease | 542 | 507 | 33 | 2 | 434 | 94 | 14 | 428 | 107 | 7 |
| Physical disease | 129 | 124 | 3 | 2 | 91 | 33 | 5 | 100 | 29 | 0 |
| Intractable disease | 21 | 20 | 1 | 0 | 15 | 5 | 1 | 16 | 5 | 0 |
| Psychiatric disease | 2 | 2 | 0 | 0 | 1 | 1 | 0 | 2 | 0 | 0 |
| Unknown | 9 | 9 | 0 | 0 | 6 | 3 | 0 | 7 | 2 | 0 |
| #4. What is appropriate first aid for a student having an epileptic seizure? (MRA) | | | | | | | | | | |
| Observe calmly | 550 | 514 | 33 | 3 | 441 | 93 | 16 | 438 | 106 | 6 |
| Secure airway | 482 | 448 | 31 | 3 | 376 | 92 | 14 | 374 | 101 | 7 |
| Call an ambulance | 277 | 263 | 11 | 3 | 225 | 47 | 5 | 220 | 54 | 3 |
| Take student to hospital | 251 | 240 | 9 | 2 | 191 | 55 | 5 | 197 | 51 | 3 |
| Call student’s name loudly | 25 | 6 | 1 | 0 | 20 | 5 | 0 | 21 | 4 | 0 |
| Hold student in arms | 11 | 11 | 0 | 0 | 7 | 3 | 1 | 11 | 0 | 0 |
| Wake student up | 6 | 6 | 0 | 0 | 6 | 0 | 0 | 5 | 1 | 0 |
| Put something into student’s mouth | 6 | 6 | 0 | 0 | 2 | 4 | 0 | 3 | 3 | 0 |
| Other | 307 | 287 | 18 | 2 | 251 | 52 | 4 | 251 | 52 | 4 |
| #5. Is it necessary to educate other students about first aid for epileptic seizures? | | | | | | | | | | |
| Yes, as an independent theme | 49 | 44 | 4 | 1 | 41 | 5 | 3 | 35 | 13 | 1 |
| Yes, as part of first aid training | 360 | **346** | 13 | 1 | 271 | 78 | 11 | 287 | 69 | 4 |
| Yes, but not enough time | 81 | 76 | 4 | 1 | 68 | 13 | 0 | 61 | 19 | 1 |
| No | 74 | 68 | 5 | 1 | 65 | 7 | 2 | 60 | 14 | 0 |
| Others | 51 | 44 | 7 | 0 | 42 | 9 | 0 | 41 | 10 | 0 |
| NA | 25 | 24 | 1 | 0 | 19 | 5 | 1 | 20 | 4 | 1 |
| #6. Do you want to attend lectures or seminars to learn about epilepsy? | | | | | | | | | | |
| Yes | 511 | 478 | 29 | 4 | 406 | 62 | 13 | 399 | 106 | 6 |
| No | 112 | 107 | 5 | 0 | 85 | 24 | 3 | 91 | 20 | 1 |
| NA | 17 | 17 | 0 | 0 | 15 | 1 | 1 | 14 | 3 | 0 |

GES, general educational school; SES, special educational school; NA, no answer; MRA, multiple responses allowed. Thick outline indicates that statistical analysis was performed in the box. Shaded cells indicate a statistically significant difference within the box. Bold, underlined number indicates a significantly higher result in the residual analysis after the chi-square test.

Suppl Table 2: Information on individual students with epilepsy (Detailed data)

|  | Total | Type of school | | | Information from medical institution | | |
| --- | --- | --- | --- | --- | --- | --- | --- |
|  |  | GES | SES | Others | Inf+ | Inf- | NA |
| Total | 1398 | 1149 | 207 | 42 |  |  |  |
| #1. Have you obtained information on this student from a medical institution? | | | | | | | |
| Yes (Inf+) | 422 | 259 | **141** | 22 |  |  |  |
| No (Inf−) | 964 | **879** | 65 | 20 |  |  |  |
| NA | 12 | 11 | 1 | 0 |  |  |  |
| #2. Does the student have any other disability? (MRA) | | | | | | | |
| Intellectual disability | 392 | 164 | **200** | 28 | **215** | 174 | 3 |
| Developmental disability | 165 | 118 | 41 | 6 | **72** | 90 | 3 |
| Physical disability | 158 | 43 | **113** | 2 | **99** | 58 | 1 |
| Psychiatric disability | 17 | 16 | 1 | 0 | 7 | 10 | 0 |
| Higher brain dysfunction | 15 | 11 | 3 | 1 | 8 | 7 | 0 |
| None | 821 | **808** | 0 | 13 | 155 | **660** | 6 |

GES, general educational school; SES, special educational school; Inf, information from medical institution; NA, no answer; MRA, multiple responses allowed. Thick outline indicates that statistical analysis was performed in the box. Shaded cells indicate a statistically significant difference within the box. Bold, underlined number indicates a significantly higher result in the residual analysis after the chi-square test.

Suppl Table 3: Nurse teachers’ knowledge of individual students’ seizures (detailed data)

|  |  | Type of school | | | Information from medical institution | | | | | | | | |
| --- | --- | --- | --- | --- | --- | --- | --- | --- | --- | --- | --- | --- | --- |
|  |  |  |  |  | GES | | | SES | | | Others | | |
|  | Total | GES | SES | Others | Inf+ | Inf− | NA | Inf+ | Inf− | NA | Inf+ | Inf− | NA |
| #3. Do you know the seizure type? | | | | | | | | | | | | | |
| Yes | 978 | 769 | **179** | 30 | **228** | 531 | 10 | **131** | 47 | 1 | 19 | 11 | 0 |
| No | 415 | **375** | 28 | 12 | 30 | **344** | 1 | 10 | **18** | 0 | 3 | 9 | 0 |
| NA | 5 | 5 | 0 | 0 | 1 | 4 | 0 | 0 | 0 | 0 | 0 | 0 | 0 |
| #4. Which type of seizure does this student have? (MRA) | | | | | | | | | | | | | |
| LOC | 500 | 392 | 96 | 12 | 118 | 235 | 3 | 64 | 20 | 0 | 10 | 8 | 0 |
| Falling | 458 | 356 | 84 | 18 | 133 | 255 | 4 | 75 | 20 | 1 | 7 | 5 | 0 |
| Neither LOC nor falling | 110 | 83 | 26 | 1 | 18 | 63 | 2 | 21 | 5 | 0 | 1 | 0 | 0 |
| Others | 229 | 162 | **62** | 5 | 44 | 116 | 2 | 45 | 16 | 1 | 5 | 0 | 0 |
| #5. Do you know the frequency of the seizures? | | | | | | | | | | | | | |
| Yes | 1073 | 856 | **187** | 30 | **215** | 637 | 4 | 128 | 58 | 1 | 22 | 8 | 0 |
| No | 251 | **227** | 18 | 6 | 38 | **183** | 6 | 12 | 6 | 0 | 0 | 6 | 0 |
| NA | 74 | 66 | 2 | 6 | 6 | 59 | 1 | 1 | 1 | 0 | 0 | 6 | 0 |
| #6. How frequent are the seizures? | | | | | | | | | | | | | |
| More than 1 per day | 56 | 14 | **42** | 0 | 5 | 9 | 0 | 33 | 9 | 0 | 0 | 0 | 0 |
| 1 per day-1 per month | 82 | 42 | **39** | 1 | **16** | 26 | 0 | **34** | 5 | 0 | 1 | 0 | 0 |
| 1 per month-1 per 2 years | 389 | 310 | 58 | 21 | **97** | 210 | 3 | 42 | 15 | 1 | 16 | 5 | 0 |
| No seizure for >2 years | 546 | **490** | 48 | 8 | 97 | **392** | 1 | 19 | **29** | 0 | 5 | 3 | 0 |
| Unsure | 251 | 227 | 18 | 6 | 38 | 183 | 6 | 12 | 6 | 0 | 0 | 6 | 0 |
| NA | 74 | 66 | 2 | 6 | 6 | 59 | 1 | 1 | 1 | 0 | 0 | 6 | 0 |
| #7. Do you know what triggers the seizure? | | | | | | | | | | | | | |
| Yes | 544 | 427 | **104** | 13 | **157** | 266 | 4 | 72 | 31 | 1 | 8 | 5 | 0 |
| No | 845 | **713** | 103 | 29 | 100 | **606** | 7 | 69 | 34 | 0 | 14 | 15 | 0 |
| NA | 9 | 9 | 0 | 0 | 2 | 7 | 0 | 0 | 0 | 0 | 0 | 0 | 0 |
| #8. Do you know how to perform first aid for a seizure? | | | | | | | | | | | | | |
| Yes | 1105 | 871 | **200** | 34 | **246** | 617 | 8 | **139** | 60 | 1 | 22 | 12 | 0 |
| No | 276 | **263** | 6 | 7 | 13 | **248** | 2 | 1 | **5** | 0 | 0 | 7 | 0 |
| NA | 17 | 15 | 1 | 1 | 0 | 14 | 1 | 1 | 0 | 0 | 0 | 1 | 0 |
| #9. Who gave you the information on first aid? (MRA) | | | | | | | | | | | | | |
| Parents | 1069 | **840** | 195 | 34 | 232 | 602 | 6 | 135 | 59 | 1 | 22 | 12 | 0 |
| Medical institution | 272 | 111 | **141** | 20 | 103 | 8 | 0 | 128 | 12 | 1 | 20 | 0 | 0 |
| Student with epilepsy | 78 | **77** | 1 | 0 | 25 | 50 | 2 | 1 | 0 | 0 | 0 | 0 | 0 |
| Others | 20 | **20** | 0 | 0 | 4 | 16 | 0 | 0 | 0 | 0 | 0 | 0 | 0 |
| #10. Do you use the *School life guidance and management form*? | | | | | | | | | | | | | |
| Yes | 88 | 76 | 12 | 0 | **57** | 17 | 2 | **12** | 0 | 0 | 0 | 0 |  |
| No | 1292 | 1060 | 195 | 37 | 199 | **852** | 9 | 129 | **65** | 1 | 22 | 15 |  |
| NA | 18 | 13 | 0 | 5 | 3 | 10 | 0 | 0 | 0 | 0 | 0 | 5 |  |
| #11. Do you use the *List for guidance of daily life in children with epilepsy*? | | | | | | | | | | | | | |
| Yes | 45 | 28 | **17** | 0 | **27** | 1 | 0 | **17** | 0 | 0 | 0 | 0 | 0 |
| No | 1336 | **1109** | 190 | 37 | 230 | **868** | 11 | 124 | **65** | 1 | 22 | 15 | 0 |
| NA | 17 | 12 | 0 | 5 | 2 | 10 | 0 | 0 | 0 | 0 | 0 | 5 | 0 |

GES, general educational school; SES, special educational school; Inf+, information from medical institution obtained; Inf−, information from medical institution not obtained; NA, no answer; MRA, multiple responses allowed; LOC, seizure with loss of consciousness. Thick outline indicates that statistical analysis was performed in the box. Shaded cells indicate a statistically significant difference within the box. Bold, underlined number indicates a significantly higher result in the residual analysis after the chi-square test.

Suppl Table 4: Summary of responses to open questions

| #1. When not using the *School life guidance and management form* or the *List for guidance of daily life in children with epilepsy*, what precautions do you take for students with epilepsy? | |
| --- | --- |
|  | Avoid leaving the student alone |
|  | Ensure the student avoids high places and/or swimming |
|  | Ensure the student uses a different colored swimming cap |
|  | Ensure the student avoids loud noise and/or bright light |
|  | Ensure the student avoids grapefruit |
|  | Ensure the student avoids intensive exercise (judo, kendo, cycling, marathon, etc.) |
|  | Discuss the student with the attending doctor |
| #2. Do you have any special considerations for students with epilepsy? | |
|  | Make a first-aid manual for seizures |
|  | Share information and formulate a common understanding with other staff members |
|  | Observe their health condition carefully |
|  | Protect the student’s privacy |
|  | Avoid leaving the student alone |
|  | Confirm drug intake |
|  | Use a different colored swimming cap |
|  | Avoid loud noise |
|  | Ensure the student uses protectors (head, knee) |
|  | Ensure the student avoids grapefruit |
|  | Ensure the student carries their emergency card |
|  | Share information with parents in writing and by telephone |
|  | Record seizures |
|  | Use cushioned covering or head equipment |
|  | Ensure the student avoids cycling |
|  | Prepare emergency medication |
| #3. Have you had any problems with students with epilepsy? | |
|  | Student suffered an injury due to their seizure |
|  | Shortage of staff for supporting the student |
|  | Difficulty judging whether a seizure is epileptic or non-epileptic |
|  | Student has attention deficit |
|  | Indifference of the student (or their parents) to their condition |
|  | Impact on other students |
|  | Lack of information from medical institution |
| #4. Do you have any ideas for how to improve the school life of students with epilepsy? | |
|  | Prepare emergency medication |
|  | Provide a first-aid manual for each student with epilepsy |
|  | Communicate with parents |
|  | Educate staff to improve their understanding of epilepsy |
|  | Enlighten students with epilepsy, and their parents, about their condition |
|  | Communicate with doctors |
